# Dignity as an empirical lifeworld construction—In the field of surgery in Denmark

**DOI:** 10.3402/qhw.v9.24849

**Published:** 2014-07-17

**Authors:** Tina Seidelin Rasmussen, Charlotte Delmar

**Affiliations:** 1 Department of vascular surgery, Aalborg University Hospital, Aalborg, Denmark; 2 Section of Nursing, Institute of Public Health, Aarhus University, Aarhus, Denmark

**Keywords:** Dignity, Denmark, nurse–patient collaboration, asymmetrical power, hermeneutic phenomenological approach

## Abstract

Patient dignity is a complex yet central phenomenon. Disrespect for dignity can mean retention of sick role, loss of self-care and control, decreased participation and therefore influence healing. At the same time, nurses have an obligation to respect dignity, and patients expect it. In clinical practice, with the focus on efficiency and economy, dignity can be compromised. The surgical patient may be particularly vulnerable to loss of dignity, when focus is solely on surgical procedure, efficiency, and productivity. The aim of the article is to describe the characteristics of the importance of dignity perceived by four surgical patients at a university hospital in Denmark. The hermeneutic phenomenological approach of Van Manen is used to analyse and interpret data collected from in-depth semi-structured interviews. The interviews explored the lived experience with two women and two men who had undergone a surgical intervention in a Danish vascular surgery department. The thematic analysis led to the basic theme: “To be an important person” illustrated by the themes: “Being a co-player,” “Over exposure,” and “To swallow the bitter pill.” The findings provide a better understanding of patient's perspective of dignity, which is characterized by a complex interaction of several factors. Nurses should be concerned with balancing expectations, values, and opinions to maintain dignity in nursing and create a common platform for collaboration. This collaboration makes it possible for patients to be involved and have a voice in relation to nursing, treatment, and administering of time even though it could be at the expense of the terms of the system.

Respecting patients’ dignity is a fundamental value in professional nursing (American Nurses Association, 2001; Australian Nursing and Midwifery Council, 2003; Guidelines for Nursing Ethics, 2013; ICN, 2006; Jacobs, 2000; Rundqvist, Sivonen, & Delmar, 2010). Maintenance of and respect for patients’ dignity gives nursing the necessary quality because being a patient can be a threat to dignity, integrity and can cause vulnerability (Irurieta, 1999; Morris, 2012; Sprinks, 2011). Dignity is defined as a goal by the WHO (2013) and is described as a human right by Amnesty International (1948).

Reviews clarify that existing literature describes dignity in general terms but there is limited knowledge on the importance of dignity in practice (Gallagher, Li, Wainwright, Jones, & Lee, 2008; Jacobsen, 2007). The phenomenon dignity appears complex and abstract and is used in different contexts; this can lead to lack of clarity on the importance of the phenomenon (Haddock, 1996; Shotton & Seedhouse, 1998; Tadd, Bayer, & Dieppe, 2002). In order to not turn dignity into pure rhetoric or a cliché in nursing practice, there is a major need for exploring the patient perspective in relation to dignity (Lin, Watson, & Tsai, 2013; Woogara, 2001).

## Background

A Danish study emphasizes “it is about creating small everyday circumstances in which patient dignity can flourish” (Hall & Høy, 2012, p. 1). By regarding the patient as a unique human being, helping manage appearance and carry own clothes, as well as motivating mobilization, we can effect a transition from the sick role to being co-responsible for own care. It is ideal but not always possible due to time pressure, reduced number of healthcare staff, and technical issues (Hall & Høy, 2012). Political decisions, financial restrictions, and lower number of staff affect nursing quality. Time pressure and reduced number of healthcare staff affect respect and dignity expressed by less time to individual nursing, neglect, and patients pressured to opt for discharge (Irurieta, 1999). A Swedish study adds that the tendency in surgical nursing is that focus is on installing new technique, “removing the diseased,” and efficiency and fulfilment of productivity demands at the expense of maintaining patient dignity (Vendlegård, Hübner, & Lindwall, 2010). The study concludes that care actions maintaining dignity in surgical nursing must ensure that focus is not solely on efficiency and productivity.

Nurses’ behaviour and respect for autonomy are care actions of major importance for maintenance of dignity. When the nurse is polite, friendly, helpful, empathic, respectful, and takes time for an individual human being, it preserves dignity. Similarly, it is of major importance that interaction is characterized by trust, confidentiality, and openness acknowledging individual needs (Berg & Danielson, 2007; Hanratty et al., 2012; Leung, 2009; Lohne, Aasgaard, Caspari, Sletteø, & Nåden, 2010; Matiti & Trorey, 2004, 2008; Whitehead & Wheeler, 2008; Webster & Bryan, 2009).

Research from the patient perspective shows that patients are vulnerable as they feel loss of control, lack of privacy, and insecurity (Baillie & Llot, 2010). An interview study of patients undergoing spinal surgery showed that there is a demand for a feeling of safety, to be treated with respect and dignity, human contact and increased possibility for support and counselling during the course of disease (Davis, Vincent, Henley, & McGregor, 2013). At the same time, patients who have undergone bypass surgery emphasize that dignity is lost if the focus of care remains only on the surgical intervention (Lang, Poon, Kamala, Ang, & Mordiffi, 2006). Matiti (2008) describes that patients perceive dignity to be maintained when they are in control and participate in decisions. If the expectation of being taken seriously and involved in one's own care are not met, dignity can be affected (Delmar, Alenius-Karlsson, & Højer Mikkelsen, 2011). A qualitative study on patients’ dignity in relation to essential needs showed that dignity is not always maintained concerning personal hygiene and dressing (Lothian & Phillip, 2001). Busy nurses, temporary staff, and being referred to as “the number of your bed” are aspects surfacing when studying patients’ perception of lost dignity (Gallagher & Seedhouse, 2002). Album (1989, 1996) also describes the bed as the patients’ most private space. Despite this, staff move in and out of the room without gathering the tasks they have to do in the room and without knocking on the door; in this way privacy and dignity is violated.

There can be differences in the perception of patients and nurses, respectively, concerning maintenance of dignity; this is compared in a Greek study. Patients often perceive that privacy and dignity are threatened in connection with personal hygiene, participation in decisions, as well as the timing of discharge; contrary to this, the majority of nurses perceive that privacy and dignity are maintained (Lemonidou & Merkouris, 2003). Similarly, Papastavrou et al. (2012) found a discrepancy between the perception of surgical patients and nurses, respectively, of human presence and respectful behaviour.

**Figure 1:**
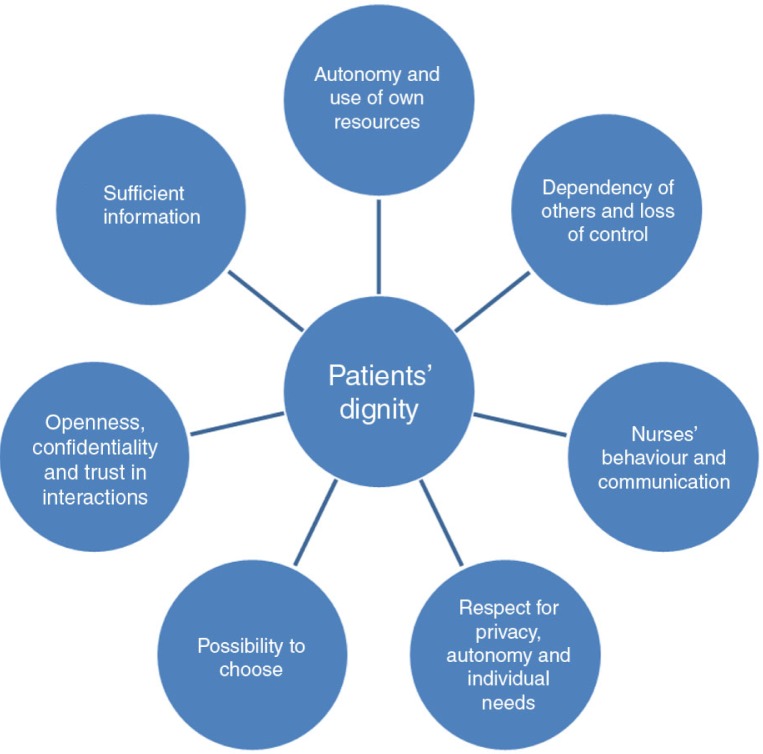
Factors affecting patient's dignity.

From a patient perspective, it is important to be treated with respect and dignity. It leads to higher satisfaction and a feeling of safety, which can impact positively on recovery and increase self-esteem (Griffin-Heslin, 2005; Joffe, Manocchia, Weeks, & Cleary, 2003; Matiti & Trorey, 2008). Care without dignity affects the patients’ recovery negatively (Brencick & Webster, 2000; Jacobs, 2000; Walsh & Kowanko, 2002). International studies have also found that patients can experience loss of control and dependency on others as a threat to dignity. Contrary to this, being involved in decisions, participating in own care, being able to choose, using own resources, and getting sufficient information can contribute to maintaining dignity (Baillie, 2009; Hanratty et al., 2012; Henderson et al., 2009; Lauck, 2009; Leung, 2009). Based on the literature review, factors affecting patients’ dignity are summarized and illustrated (Figure 1).

It seems important to maintain patients’ dignity as a lack of respect for dignity can lead to maintenance of the sick role, lost self-care, reduced involvement in decisions, as well as delayed recovery. Knowledge on patients’ perception of loss of dignity is scarce—especially in relation to surgical departments in Denmark. Therefore, the aim of the article is to describe characteristics of the importance of dignity perceived by four surgical patients at a university hospital in Denmark.

## Methodology and methods

Van Manen's hermeneutic phenomenological approach is chosen, as an approach to the empirical material, as the purpose is to make the description lively with focus on the important meaning of a phenomenon (Van Manen, 1990). The analytical approach of the study is the four existentials: space, body, time, and relations, which together form a coherent whole—the phenomenological lifeworld. Using existentials as a horizon of thought, it is possible to describe the lifeworld and experiences in both a detailed and specific manner.

### Clinical context and selection of informants

The study is a descriptive pilot study conducted at a unit for vascular surgery at a university hospital in Denmark. The unit has 10 beds and treats patients for diseases in the arteries except in the heart and brain. The majority of patients are above 60 years of age, and a large number of patients have comorbidities such as diabetes, Chronic Obstructive Pulmonary Disease (COPD), reduced kidney function, and heart disease. The patients’ disease and surgical intervention in legs, stomach, neck, and groin region often mean that the patient is dependent on help, for example, personal hygiene, wound care, and mobilization. To capture nuances and depth in the empirical material, a purposeful sample is made based on variation in age, gender, course of admission, and experience in being dependent on others. To obtain validity, the study emphasizes capturing nuances and depth of the lifeworld of each individual narrative instead of broadness and number of informants. This is motivated by the qualitative lifeworld approach (Dahlberg, Dahlberg, & Nyström, 2008; Kvale & Brinkman, 2009) also in line with Van Manen (1990).

Participants are required to speak and understand Danish as it is assumed that expressions would be influenced if the interview involved a translator. To maintain confidentiality, the participant must be able to be transferred to an undisturbed location. Not being oriented in time and space or having complications makes it ethically irresponsible to include the patients. Thus, the study included four informants, two women and two men, between 53 and 74 years of age. The patients had undergone surgical interventions, had different experiences with hospital admissions, and were dependent on help from others (Table I).

**Table I:** Participant characteristics.

Participant	Gender	Age	Experience with dependency of others	Hospitalization period in days/day for interview
1	Male	61	Previously received help for a prolonged period of time in his own home, now self-sufficient Two previous admissions	10/7
2	Male	53	Self-sufficient No previous admissions	3/3
3	Female	70	Receives full help in her own home Multiple previous admissions	29/23
4	Female	74	Previously received help for a shorter period of time in own home Four previous admissions	9/5

### Data collection

In-depth qualitative research interviews were selected to collect narratives, as the purpose is to understand the lifeworld from the informant's point of view and to unfold the perceived meaning attached to their experiences (Dahlberg et al., 2008; Kvale & Brinkman, 2009). The interviews with semi-structured questions were conducted in February and March 2013. The first contact was the contact nurse. The informant had the option to determine if he/she wanted to participate in the study. Subsequently, contact was established with the interviewer. The interviewer explained the purpose of the study and the patient's rights. Based on oral and written information about the study, the informant would have to decide whether to participate or not by signing a consent form.

The interviews were performed in an undisturbed place, lasted between 45 and 60 min, and were tape-recorded. Subsequently, they were transcribed verbatim respecting language expressions and nuances as Van Manen (1990) sees language presentation as substantial and a factor considered as a meaningful aspect. The interview was conducted using an interview guide. The open questions in the interview guide were based on knowledge of dignity acquired on the basis of the initial literature review and knowledge documented in the scientific literature. It was deemed necessary to obtain spontaneous and comprehensive descriptions from informants as the phenomenon dignity can appear abstract and complex. Focus was on whether questions in the interview guide were meaningful to the informants. An experienced interviewer listened through the first interview to assess if the interview facilitated rich description from the participant. This led to a few alterations in the formulation of questions increasing focus on making room for breaks.

The purpose of the study and informants’ rights were explained in the beginning of the interview. After initial “small talk,” the informants were encouraged to tell in detail about their experiences of being admitted. Then the informants were asked, for example, about their possibilities and rights, involvement in and influence on decisions, as well as the nurses’ attitude to them as patients. At the end of the interview, they were asked to describe their dignity and elaborate on what could maintain their dignity or prevent maintenance of dignity during hospital admission. During the interview, there was focus on pursuing interesting themes cropping up. Interview knowledge is an asymmetrical power relation as the interview is defined and instrumentalized by the researcher. It is important that the researcher reflects on the significance of this power in relation to the knowledge produced (Kvale & Brinkman, 2009, pp. 50–52). During the interviews, it has been attempted to follow the participants’ narratives and let them finish talking before asking any elaborating questions, showing respect for the participants’ experiences and not the pre-understanding of the researcher. Also, during the analysis, the texts have been carefully considered if interpretations are rooted in the participants’ narratives.

### Data analysis

Data analysis followed the principles of Van Manen (1990) and was step-wise at two levels. *The vertical level* where each interview was studied individually and *the horizontal level* where associations between interviews were studied. Existentials were guiding the process (Van Manen, 1990).

### The vertical level

Level 1 provides a holistic understanding of the individual patient's experiences. In this phase, the transcribed interviews were read several times and condensed to a shorter description of the informant's experiences of the importance of the phenomenon dignity.

Level 2 is a selective approach. The data material is studied to select sentences and meaning units appearing essential in relation to themes of importance to the phenomenon dignity. A total of 16–27 themes were found in each interview. Metaphors were studied as language images can contain important meaning.

Level 3 is a detailed study with the purpose of capturing the professional understanding of a phenomenon. Distinctions are made between essential and non-essential themes. As an example, it was an essential experience “to be allowed to be who I am.” The question investigated in the data at this level was: How does this sentence/paragraph illustrate the informant's experiences of the importance of dignity in a nursing professional perspective? Pre-understanding is consciously included at this level to find essential themes.

### The horizontal level

At level 4, essential themes from the individual interviews are compared and presented in a main theme and sub-themes common to informants. This meant that all interviews were read again but now as an entirety as the intention was to obtain a better understanding of the common experiences. Whether common themes were essential was determined by asking the questions: Is the experience of dignity the same without this theme? And does the experience of dignity lose its basic meaning without this theme? Existentials were guiding the process and ensuring that the analysis was closely related to the common human aspects of the lived experiences. To follow the recommendations of the methodology balancing between parts and entirety, the horizontal themes were compared with the vertical themes to validate previous interpretations. Pre-understanding was actively at play in a professional discussion, nuancing the meaning in the individual statements to obtain a new understanding of the phenomenon. During the process, there was an understanding that pre-understanding could be a hidden tyrant taking away the power from openness and true understanding (Eilertsen, 2000). To enhance trustworthiness, interviews and the analysis and interpretation process were consecutively discussed with a very experienced researcher. In addition, the interviewer had knowledge of the department where the informants were hospitalized, whereby the pre-understanding might influence the findings. However, it was seen as an advantage to be aware of the context in which the informants’ narratives emerged.

### Ethical considerations

The study was presented by phone to the ethics committee in the North Denmark Region (2012) confirming that the study did not need approval as it does not comprise biological material. The study includes sensitive personal data and approval was thus obtained from the Danish Data Agency (2012); CVR-nr. 11-88-37-29. Journal number: 2012-41-0222. The Ethical Guidelines for Nursing Research in the Nordic Countries (2003) and rules on confidentiality were followed. It was clarified to informants that data were stored safely and deleted after the completion of the study (Eilertsen, 2000). Moreover, informants were anonymous in notes and transcribed interviews. Informants were told they could withdraw consent without any consequences for their hospital stay.

## Findings

The analysis lead to the common basic theme—*To be an important person* illustrated by the themes: *Being a co-player, Over exposure*, and *To swallow the bitter pill*. Overall, the themes give an impression of what characterizes the phenomenon dignity based on patients’ experiences. The thematic division is, however, an empirical construction and the themes do not have a clear delimitation. They are interwoven, which will be evident in the subsequent discussion.

In this study, it appears important from the patient's perspective, to be respected and acknowledged as an important person, assuming an active role as an equal co-player in care and treatment. Patients would like to be involved in decision and be accepted for what they can contribute with. This means that involvement in decisions is context-dependent, depending on the patient's situation, wishes, and expectations as well as how the collaboration with the nurse works.

The analysis also shows that respecting privacy is important to uphold dignity. But there could be a schism when usual boundaries concerning privacy are shifted during hospital admission. Patients could overstep natural boundaries for privacy when a special understanding with fellow patients occurs. However, the community with fellow patients cannot stand alone if dignity is to be maintained, as patients experience information from nurses about care and treatment to be of major importance to dignity.

### Being a co-player

The theme concerns experiences on co-determination, respect, acknowledgement, and rights in the collaboration with the nurse. To be a co-player is described as being an important person as you are acknowledged as a whole person and “not just a piece in a bigger game” (Participant 1). This was expressed when the nurses, despite being busy, were there for the individual. It is pronounced in the maintenance of dignity as the patient is noticed and respected as a person worth spending time on. Informants express understanding of the busyness and that this is communicated so that they know what to expect. In this way, the patient is a co-player understood as having a joint responsibility for the day to run smoothly. Participant 4 says: “you are involved and it is okay to wait a little longer.” Another perspective is revealed when the lack of time means the patient is rushed and denied the possibility to participate in the care at his or her own terms. It is a threat against dignity. Participant 3 tells:

You have to be ready, the doctor is coming. Who says I have to lie there and be ready? That is where I draw the line, I decide. I get insulted and loose my temper. I know I am slow but let me be slow—I will be clean and neat in my own way. It is difficult when you are not allowed to be who you are; I take pride in doing what I can.

This lack of respect for rights, co-determination, and participation in own care and treatment is also described by participant 4. She has had diabetes for many years and thinks about eating a diet with no sugar. At the hospital she is told she can eat all food and use highly effective insulin as a supplement:

It is my choice—I do not dare to eat it. The nurse was getting tired because I wouldn't do as she said. It is not well received if you don't take good advice and think about what to do your own way. This is respect for the knowledge I have brought with me (Participant 4).

Participant 1 adds: “Don't act too wise or have an opinion on things—then you get unpopular.”

The empirical data show that the patients wish to be co-determinants and be acknowledged and shown respect for what they can offer. Participant 2 compares collaboration to a marriage where you do not necessarily agree but have to respect the other person's attitude if the dignity is to be maintained. Contrary to this, the empirical material also shows that the demand for co-determination is not always present. This is seen when the disease leads to lack of energy to make a choice or when trusting the expertise of another means you do not want to interfere. Participant 4 puts it like this:

Well, I felt like this all the time; I felt like it was not me deciding anything about that leg, right? And I don't want to either because they have the expertise and the necessary knowledge. The doctors have told me we do this and we do that and then they should just do it. I feel safe. I don't put up an ultimatum, now I want to decide which way to go … no, actually I don't wish that—I'm completely confident about them doing the right thing and yes it's okay with me that they take over—I have worries enough if you see what I mean.

It appears that when given a choice, you feel involved in care and treatment and you feel in control of what is going on; this contributes to maintaining dignity. There seems, however, to be a tendency that informants did not feel involved as much as they expected. Participant 1 who is not allowed to manage his own medication describes this: “I'm surprised that they have had my medicine in their cabinet. They probably need to watch what I'm taking. Well, I am a patient but there is nothing wrong with my head; I could manage that.”

The possibility to be involved in decisions is linked to the information the patient receives. The empirical data show that information provide security and reduce worries, and the patients perceive their dignity is maintained because they understand the thoughts behind what is happening. In this way, the patient feels important and is capable of being a co-player because he/she is involved in making the decision that is best for him/her. The opposite is seen when participant 3 tells about the experience of going to do her exercises without anybody discussing the timing with her: “Tell what is going on. It is possible that I might not be able to make it today. It's all about getting a little control of the situation—to have the possibility to choose what I prefer to do today.”

To perceive that you get the information you want is linked to the relationship with the nurse as the patient depends on her being there to tell what is going to happen. At the same time, the relationship with the nurse means more information and it becomes evident that it is important for the perception of dignity that the collaboration is well functioning and characterized by mutual respect. Patients make an active effort to ensure that the collaboration remains positive. Informants stress how humour is used, how they avoid frequent disturbances, how they help and feel responsible for maintaining a good friendship when there has been a disagreement. The following examples illustrate this:

I want to be one they like, one they want to visit and chat with. I'm very conscious of not calling them all the time. If you disturb them for no reason they could see you as a burden. You worry about that because you are dependent on them; so I hold back and collect things (Participant 1).

Participant 3 adds: “My temper can get the better of me and I say something rude. But I make sure we always stay good friends.”

Concerning the collaboration, it is also stressed that the behaviour of the nurse is important for the perception of respect and maintenance of dignity. It is stressed that nurses introduce themselves, listen, smile, show empathy, are helpful, and know how to ask about the difficult things without the patient mentioning it. The ability nurses have for “small talk” is particularly valuable because it shifts focus from the unpleasant in a situation. Participant 2 explains: “It is rare there is an awkward pause—just talk about this morning's news. So the thoughts are distracted from her helping you to get washed or whatever.”

The good atmosphere at the department is mentioned in particular when the patient feels that the nurses like their job and want what is best for the patients. This has a positive effect on the atmosphere in the department and on the perception of dignity.

### Over exposure

The theme includes the experience that it can be necessary to adjust dignity when you are admitted to undergo surgery. When needing help, it is perceived necessary to adjust by making compromises concerning privacy and dignity: “It turned out that when I needed help it is the same for everybody. There is no choice if I want to be helped I have to adjust to the framework and take the consequences that follow” (Participant 2).

Participant 1 adds: “When you are helped it affects your pride. You can feel embarrassed, uncomfortable, yes humiliated and helpless. Almost like a baby again but it is necessary for a while.”

Patients accept the hospital as an unfamiliar framework where the personality can be lost for a while. It is a framework where you accept that “strangers get close” (Participant 2) and that “you are visiting somebody you don't know” (Participant 2). Participant 1 describes it as placing the dignity under your pillow, as it is easier not to think about dignity when it has to be adapted rather often. The perception of adapting and adjusting dignity is expressed when showing the body to the staff but risk exposing the body to other patients. Nurses make an effort to respect privacy and minimize exposure of the body and the unpleasant feeling linked to this. It is described how curtains are used, how patients are encouraged to wear their own clothes, and how nurses make tasks feel natural. When nurses have a relaxed attitude to the task it is easier to manage it. This is illustrated by the description of a wound inspection in the groin:

They pull your pants down to inspect the wound. It is rather strange because it's not somebody I know and then looking inside your pants. But to her it's rather common and you can sense that. They see other peoples’ bodies all the time (Participant 1).

Participant 3 describes a similar situation. She could not control her stools due to treatment with antibiotics, and it was running out on the floor. It is illustrated how she got through this uncomfortable experience with help from the staff.

It was embarrassing and I felt so uncomfortable. But they didn't turn their noses up or get mad. I thought show me the mouse hole where I can crawl in. When their spirits are high and you feel the good atmosphere then you get through it with your dignity intact.

It was also noticed that when curtains are forgotten and other patients can see what is going on, it seriously violates the dignity. Participant 4 describes the episode with wound inspection at rounds: “I was lying in the bed and then they pack it all out. Everybody could see what was going on and I cringed because, honestly, it looked rather disgusting.”

The data show that not only the patient but also fellow patients feel uncomfortable when curtains are not drawn and you risk seeing something that is none of your business. The physical framework is in this connection described as part of the reason why it is not always possible to protect the dignity. Patients in rooms with more beds may experience exposure by listening to what is said. This is seen in an episode where an accident with urination in the bed is discussed behind the curtain—but loud enough for the others in the room to listen in. In another situation, a patient is informed during rounds that her leg might not make it. It is uncomfortable for the patient when others can listen in as the patient feels exposed and “you can feel the assumed pity of others” (Participant 3). On the contrary, the patient's own situation with lack of energy to be involved can make it difficult to ask questions about what you hear. It will “hang in the air” (Participant 4)*—*understood in the sense that you feel you ought to ask elaborating questions.

There is, however, an opposite perspective to this. It is seen that the hospital is perceived as a room where it is allowed and meaningful to talk about private issues with people you do not know without perceiving this as exposure. Shyness about intimate conditions can lose its importance as you experience that you are being met by fellow patients with a special understanding; an understanding which contributes to maintaining personal dignity during the admission. Participant 3 explains: “Normally, I keep such things to myself. But here it's accepted that accidents can happen. Then I talk to the ladies in the room about it. Then it is over and done with and I'm not sad anymore.”

Participant 1 adds: “When I walk around the room in my underwear they don't care. They know you can have one of these days where you don't feel like doing anything.”

Another nuance is seen in Participant 1's description of how sharing things with each other can make you feel safe:

We shared our worries and I talked about how concerned I was about losing my leg. We were here together and it became natural to use each other. It feels safe to have someone to share things with. I don't have my family close by so I need someone to talk to.

Patients can in this way use each other as a tool to protect their dignity. You can be confident with fellow patients so you don't feel exposed when the body's reaction to care and treatment is expressed as exposure, lack of control of bodily functions, or when you listen in on personal information.

Hospital clothes are also a cause of exposure especially when it does not fit very well and when the patient has to leave the department. At the same time, it is felt that hospital clothes make you anonymous. This is described by participant 2 who wears his own clothes:

It has to do with the personality—now I'm myself again. You look very different in here. When you are a patient, personality is peeled off; it's a little bit like being a prisoner in the striped uniform and a hat; then you are anonymous.

Choosing to wear own clothes can thus be a conscious choice to maintain personality and to protect the dignity. Regardless of choice of clothes you are still accepted as a part of the patient community. Participant 1 explains: “Poul always wears his own (clothes) but he is still one of us.”

### To swallow the bitter pill

The theme includes experiences wherein it can be necessary to set aside your privacy when patients share a room and have to adapt to hospital rules and framework. Based on the data, it is evident that privacy is highly affected during hospitalization. Participant 3 experiences that privacy does not exist even though it is important to the individual person. Participant 2 says the following about privacy:

In a room with more people, dignity is mostly affected. Just the television—we are more to decide how loud it should be and what to watch. And as a patient it happens that you have to break wind. It is close so others can hear it. This is exactly when dignity is lost, yes it gets into play.

Focus on privacy is demanded but at the same time, patients are realistic about the difficulties involved in observing respect for privacy within the framework the hospital offers. It is mentioned: “It is an older house, this is how the framework is” (Participant 2) and “The walls are thin so the sounds are in the room” (Participant 1). Participant 3 mentions that there are only a few single rooms and they are used for those who need them the most. At the same time, it is seen that it is demanding to share a room with others. The demand to be social and participate in what is going on in the room can be hard when you are ill and do not have the energy. There is a dilemma when the patient appreciates having a community with fellow patients but also experiences the need to be alone. To be in this dilemma is described by participant 2 who needs quietness and draws the curtain. It gave a temporary feeling of guilt towards fellow patients as it is described as “dropping out of the community” (Participant 2). The experience of being a part of the community is evident when participant 1 compares the community in the room with a crew:

We are in the same boat and we are going through the same. You can say we are the crew making sure the boat arrives to the harbour safely. Because we stick together in the room; we at least try to help each other arrive safely.

This experience of having a community can be connected with the shift in boundaries concerning privacy happening during admission. In the previous section, it was described how participant 3 and participant 4 found it meaningful to talk about private issues with people they did not know. To come to terms with being close to others is described as being easier when “you fit a little together” (Participant 2). In such a situation, it is actually seen that it is an advantage as you help and support each other. Another nuance is seen when it is perceived as transgressing to be in a room with people who are very different from yourself:

A totally different type can give rise to conflict. You have to swallow a few pills because we have to stay friends. Take my fellow patient—he is a special guy, very demanding. He fiddles with tubes and yells at the nurse. He is opposite me as a person. (Participant 2)

The data reveal that there are some bitter pills to swallow when you are close to people who do not necessarily have the same personality. Another perspective is seen when it is necessary to *swallow bitter pills* in collaboration with the nurse. This is experienced when rights and habits are not respected. This is reflected in the following experiences on how patients can be denied the right to decide about their daily rhythm.

You can't go to bed early because you need to take your medicine at 22.00. Then they wake you up to take the pills and I don't think that is okay. And no kidding, then you are woken up again before 6 o'clock. Actually, I have tried to be woken up by the light from a flashlight in my head (Participant 3).

It has happened that I have done something even though I didn't want to. Some say you have to go to bed before the nightshift arrives. I didn't like that then I would toss and turn. That is not respect. They have to respect my daily rhythm because I only sleep 5 h (Participant 4).

The experiences are described as unorganized behaviour which is difficult to accept and it is hard to find the purpose. This leads to feelings of lack of respect and understanding for personal needs. Another variation in connection with rights is seen when it is experienced that the patient has not been informed about changes in the medical treatment. Participant 1 has experienced asking for less painkillers. He was told that this was already an ongoing process and that he had been given a salt tablet several times. This was perceived as very insulting as he felt cheated, looked down at, and there was a breach of trust.

## Discussion

The section discusses the collaboration with the nurse focusing on co-determination, respect, acknowledgement, and rights as well as privacy knowing that other perspectives in the analysis are in play. These perspectives seem to be particularly prominent in relation to patients’ perception of the importance of dignity and thus makes the discussion topical.

The findings show that patients have a clear perception of the factors affecting dignity. At the same time, they have expectations to how to maintain dignity. It appears important from the analysis to be respected and acknowledged as an important person. It is underlined how the patient wishes to be a co-player and be involved in care and treatment assuming an active role. This highlights a shift from previous perceptions of the health professional taking on the expert role and the patient assuming the passive role as receiver of care and treatment (Martinsen, 1993, 2006). Related to own practice, it is experienced in nursing today that there is an increased focus on motivating patients to self-care understood as self-help, joint responsibility, and co-determination. It appears from the findings that this is appreciated by the patients as involvement and co-determination is perceived as contributing to feeling more in control of what is going on as a patient, which contributes to maintaining dignity. Studies report, however, that increased focus on independence of help and co-determination in nursing can inspire to an inappropriate perception of the patient. The patient can thus be perceived as constantly being active, capable of helping himself/herself, and making a choice (Delmar, 1999). Another variation related to co-determination in this study is that the demand for co-determination does not exist in all patients and some disease situations can lead to the wish for other people to take over. Other studies have reported that not all patients wish to be offered or are capable of managing a choice (Välimäki et al., 2004; Veatch & Fry, 1987). Nurses should thus focus on the individual human being to avoid misinterpretation of co-determination and independence in the interaction. In this way, the wish to help the other can be repressed by the demand for self-care and dignity can be lost.

The nurse has a responsibility for the management of self-care as described by Scheel (2005). She expresses that self-care is necessary in nursing but there is a danger that self-care includes an oppressive and amoral way of acting if standing alone or becoming an ideology. If self-care is the goal alone, the nursing becomes a “take care of yourself theory” focused on the independence of the individual without considering the context of the individual. Scheel (2005) argues that self-care and care should be seen in a connection and that the nurse has an ethical responsibility to the patient. She says that nurses can act irresponsibly ethically speaking if the only focus is to meet the expectation of others to self-care and faster discharge. In such cases, nursing is reduced and repressed in the space where care is unfolded. Martinsen (1989) adds that self-care in nursing can be excluding as the perception of self-help and independence as leading values will exclude the weak and those needing care the most. From this it is clear that co-determination and involvement in care and treatment should take the starting point in the individual patient and his/her situation if dignity should be maintained. This is illustrated, for example, by participant 2's metaphor of the patient–nurse collaboration as a marriage where it is decisive for the perception of dignity that the attitude of the other part is respected.

The findings show that patients adapt and adjust personal dignity during hospital admission. According to Nordenfeldt (2004), humans will sooner or later experience loss of dignity, as age and physical health are not permanent. An interesting perspective in relation to this is described by Edlund (2002) who finds that when dignity can no longer be obtained, another dignity is allowed to be important. In this way, you reconcile with the situation you are in and the perception of dignity returns. This shows that the strive in humans to be important and worth something; in relation to this study this is seen in the overall theme *To be an important person*. Therefore, nurses should help patients maintain dignity or support patients that a new dignity will appear and become important. This demands collaboration to be characterized by trust.

It is very important to the perception of dignity that the patient–nurse collaboration is perceived as well functioning and characterized by trust. The findings emphasize how patients have many and different thoughts about maintaining collaboration as the patient is dependent on the nurse's help and information to avoid dignity being threatened. The patients help, are conscious not to disturb, use humour, strive to be a person the nurse likes, as well as feeling responsibility for becoming friends again after a disagreement. The literature does not contain many descriptions of the considerations and the active effort patients make to contribute to maintenance of the good collaboration. Others find that patients hold back uncomfortable experiences and express that ideal nursing is not possible due to busyness (Baillie, 2009; Henderson et al., 2009).

Empirical statements highlight vulnerability in connection with the internal power structures. The asymmetrical power structure in the interaction as the patient is dependent on the nurse has previously been described by Delmar (1999). It is therefore not surprising that the asymmetrical power structure appears in these data. A pre-requisite for maintaining patients’ dignity must be awareness of the vulnerability which is part of the people living with a disease and the awareness of the power structure to use it to the benefit of the patient (Scheel, 2005).

Due to the power structure in the interaction, nurses have a moral responsibility and a claim to welcome the other to maintain dignity (Scheel, 2005). According to Løgstrup (1996/1971), any meeting involves daring to take a step forward and is equal to exposing yourself and making yourself vulnerable, as there is a risk that the trust is rejected. If you are rejected with indifference and lack of kindness, distrust will grow. Løgstrup points out that human existence is a mutual dependency of each other and that we cannot ignore the power at play in relations. The ethical demand is to let the power serve the other which is similar to acknowledging that power is a part of all relations (Løgstrup, 1996/1971). Furthermore, Løgstrup points out that the demand can never be managed by taking away the independence for the other person's sake. In the light of Løgstrup's thoughts, it is made clear how it can influence dignity when the ethical demand is not managed and own rights acknowledged. This is illustrated when participant 3's wish to take care of personal hygiene was not acknowledged and she expresses being chased and not being allowed to be the person she is.

Similarly, participant 4 does not perceive that her own knowledge, responsibility, and independence to manage diabetes are acknowledged. She feels her respect and dignity are violated. Instead, both informants experience that they are difficult and not willing to cooperate, which leads to negative interaction with the nurse. A similar finding was seen in Chochinov (2007) as patients perceived as being unwilling to collaborate can experience a condescending and a distancing attitude and behaviour from the nurses. When patients are perceived as difficult and not being willing to collaborate, it can be caused by a clash between nurses’ and patients’ expectations, values, and opinions on dignified nursing (Baillie, 2009; Chochinov, 2002).

Respecting privacy is important to dignity, but the findings shed light on a dilemma. On the one hand patients demand respect for privacy and it is mentioned that when privacy is violated then dignity is violated. That respect for privacy is important for the experience of dignity is comparable to previous findings (Album, 1996; Jacobs, 2000; Randers & Mattiasson, 2004; Widang & Fridlund, 2003). On the other hand, Isaksen and Gjengedal (2006) describes that the norm of the culture is that privacy must not be violated, as this is something other people do not have the right to step into. He argues that privacy can be difficult in a hospital because of the presence of other patients and it is seldom possible to withdraw and give the disease less attention. This is also reflected in the findings when it is emphasized that it is demanded to be social and participate when you share a room with other patients and the physical framework makes it hard to be private when you can see and hear what is going on. The present study showed that there could be a schism when usual boundaries concerning privacy are shifted during hospital admission. The findings make it visible how it can be perceived as natural to overstep the boundaries of privacy when it is meaningful and there is a special understanding sharing private issues with fellow patients you do not know. The perception of own body, the presence at the hospital, the hospital room, and the relation understood as the community with fellow patients also makes it acceptable to just walk around in your underwear even though it was not an option according to the usual norms for privacy. The study shows that nurses make an effort to respect privacy within the given framework, which has been studied by Lawler (2003/1991). She has explored how nursing can be organized to avoid violation of privacy. She describes the importance of nurses protecting patients’ sense of embarrassment understood as violation of shyness when lack of control of the body is experienced during admission. In these situations where patients need help or when bodily functions are out of control, nurses help patients make the situation bearable.

In relation to the schism in this study, it is questionable if the nurse should always consider the body as a private thing for the patient's sake. Our findings showed how patients use each other as a tool to protect dignity. You acquire a sense of confidentiality with your fellow patients and do not feel exposed or that privacy is violated when the body's reaction to care and treatment is expressed as, for example, not being able to control stools or not have the energy to put anything on but underwear.

Lomborg (1994) has also studied this in her criticism of Lawler's thoughts. Lomborg points out that it can be time to move away from being controlled by privacy. However, she also talks about making a social room to de-privatize the body of hospitalized patients. She suggests gathering patients with the same diagnosis and problems to create a concept where the importance of privacy is reduced. At the same time, Bäck and Wikblad (1998) reported in a study of nurses’ and patients’ perception of the need for privacy during admission that nurses find it more important than the patients to protect privacy. This is in line with the findings of the analysis showing that patients can experience that the importance of respect for privacy is reduced when finding a community and feeling safe with fellow patients and in this way help each other maintain dignity during admission. Album (1996) supplements this reporting that through exchange of experiences and perceptions the patients gain control of their situation as it becomes transparent what you have been through and what to expect. As a consequence of spending time together during admission, patients will take an active part in each other's lives. Album adds that they often talk about issues of a health-related and intimate nature because they find a common understanding (Album, 1996). At the same time, fellow patients are perceived the most credible source of information as they contribute with knowledge on the basis of lived experiences (Album, 1996).

The findings also show, however, that informants experience that information about care and treatment from nurses is of major importance to dignity, as you feel involved. Therefore, it must be emphasized that community with fellow patients cannot stand alone if dignity is to be maintained.

## Conclusion

The aim was to illuminate, that it seems important to maintain patient's dignity, as the lack of respect for dignity can lead to maintenance of the sick role, lost self-care, reduced involvement in decisions, as well as delayed recovery. Knowledge on patients’ perception of loss of dignity is scarce—especially in relation to surgical departments in Denmark.

The findings showed that patients’ perception of dignity is characterized by a complex interaction of several factors. Patients have a clear perception of the factors affecting dignity. At the same time, they have expectations on how to maintain dignity. It appears particularly important, from the analysis, to be respected and acknowledged as a person of importance. It is underlined, how the patient wishes to be a co-player and be involved in care and treatment assuming an active role. This highlights a shift from previous perceptions of the health professional taking on the expert role, and the patient assuming the passive role as receiver of care and treatment.

Respecting privacy is important to dignity, but the findings shed light on a dilemma. On the one hand, patients demand respect for privacy and it is mentioned that when privacy is violated then dignity is violated. The present study showed that there could be a schism when usual boundaries concerning privacy are shifted during hospital admission. The findings make it visible, how it can be perceived as natural to overstep the boundaries of privacy, when it is meaningful and there is a special understanding sharing private issues with fellow patients you do not know—a special kind of understanding which leads to patients helping each other to maintain dignity during admission. The findings also show, however, that informants experience that information about care and treatment from nurses is of major importance to dignity, as you feel involved. Therefore, it must be emphasized that community with fellow patients cannot stand alone if dignity is to be maintained. Trust and mutual respect in collaboration with the nurses is important to dignity. It is a key factor that nurses are conscious professionally about how to manage power structures in the meeting to the benefit of the patient. Nurses should constantly be concerned with balancing expectations, values, and opinions to maintain dignity in nursing and create a common platform for collaboration. This collaboration makes it possible for patients to be involved and have a voice in relation to nursing, treatment, and administering of time even though it could be at the expense of the terms of the system.

## Limitations of the study

The question of applicability to other surgery settings in Denmark is essential. The answer to the question of what counts as applicability will be that “generalizability” in qualitative research builds on recognizability and challenges to practice (Delmar, 2010). Recognizability appears by looking for communalities, similarities, and differences. But this can only form part of the “generalizability” of a finding; knowledge should be recognized and confirmed by others. Only when the recipient of new knowledge is able to relate it to his own practice, only then makes it sense to him and the road is clear for understanding and practical application of the knowledge (Delmar, 2010).

This is a pilot study with four participants which means that data saturation is not obtained. Even though we have highlighted new knowledge, further research in the field of surgery has to be done.
